# Supplementary material for: MetaRibo-Seq measures translation in microbiomes
Source: Nat Commun. 2020 Jun 29;11:3268. doi: 10.1038/s41467-020-17081-z (PMC7324362; doi:10.1038/s41467-020-17081-z)
Supplement: Supplementary file 10 — Supplementary Data 7 [file 41467_2020_17081_MOESM10_ESM.zip › File2/Confidence_High_Taxonomy/133045_out.krona.html]

Javascript must be enabled to view this page.

members
magnitude
magnitudeUnassigned
count
unassigned
taxon
rank

133045\_out

2

2
2
superkingdom

phylum
1239
1

class
1
186801

186802
1
order

family
541000
1

1
2048137
genus


SRS013940\_contig\_number\_contig-100\_42897.42898
species
1628085
1

phylum
204428
1

204429
1
class

51291
1
order

1
809
family

810
1
genus

species

SRS012849\_contig\_number\_34315
813
1
